# Pulsed Electric Field and *Salvia officinalis* L. Leaves: A Successful Combination for the Extraction of High Value Added Compounds

**DOI:** 10.3390/foods10092014

**Published:** 2021-08-27

**Authors:** Vassilis Athanasiadis, Achillia Lakka, Dimitrios Palaiogiannis, Vasileios M. Pappas, Eleni Bozinou, George Ntourtoglou, Dimitris P. Makris, Vassilis G. Dourtoglou, Stavros I. Lalas

**Affiliations:** 1 Department of Food Science & Nutrition, University of Thessaly, Terma N. Temponera Str., GR-43100 Karditsa, Greece; vaathanasiadis@uth.gr (V.A.); achlakka@uth.gr (A.L.); dipaleog@med.uth.gr (D.P.); vpap@uth.gr (V.M.P.); empozinou@uth.gr (E.B.); gntourtoglou@uniwa.gr (G.N.); dimitrismakris@uth.gr (D.P.M.); 2 Department of Wine, Vine, & Beverage Sciences, School of Food Science, University of West Attica, Ag. Spyridonos Str., GR-12243 Egaleo, Athens, Greece; vdourt@uniwa.gr

**Keywords:** pulsed electric field, *Salvia officinalis* L. leaves, extraction optimization, polyphenols, green extraction

## Abstract

The present study aimed to evaluate the pulsed electric field (PEF)-assisted extraction of phytochemicals from *Salvia officinalis* L. leaves. The study parameters included a PEF pulse duration of 10 or 100 μs for 30 min, using different “green” extraction solvents: pure ethanol, pure water, and their mixtures at 25, 50, and 75% *v/v* concentrations. The resulting extracts were evaluated against reference extracts obtained without PEF. For estimation of the extraction efficiency, the content in total polyphenols, individual polyphenols, and volatile compounds, as well as the resistance to oxidation, were determined. The optimal PEF contribution on the total and individual polyphenols, rosmarinic acid, extractability (up to 73.2% and 403.1% increase, respectively) was obtained by 25% *v/v* aqueous ethanol solvent using a pulse duration of 100 μs. PEF was proven to also affect the final concentration and composition of volatile compounds of the extracts obtained.

## 1. Introduction

*Salvia officinalis* L. (garden sage) is a Mediterranean native perennial, evergreen aromatic subshrub, belonging to the family of Labiatae/Lamiaceae. The plant serves as an aromatic agent (food flavoring, cosmetics industry) and a medicinal plant [1,2]. The flowers, leaves, and stems are the main parts of pharmaceutical importance [1], with the leaves being the most interesting both for the medicine and food industry. *Salvia officinalis* L. leaves (SOLL) contain a vast phytochemical amount [3]. Sharma et al. [4] reported 160 polyphenolic compounds including caffeic, rosmarinic acid, quercetin, and other flavonoids and phenolic acids. Mono-, di- and tri-terpenes (1,8-cineol, carnosic acid, carnosol, or ursolic acid) are also known to be contained in SOLL. The composition varies depending on the locality, seasonality, extraction solvent, and chosen extraction procedure [5].

For the production of SOLL extracts, several techniques have been thoroughly investigated. These techniques include maceration, ultrasonic-assisted extraction, microwave-assisted extraction, supercritical fluid extraction, isolation of volatile compounds, and Soxhlet extraction [6,7,8,9,10,11]. However, the negative environmental impact that accompanies reduced extraction selectivity and the thermal decomposition of sensitive phytochemicals, as well as the high cost and energy demand of the above technologies, reveals the need for greener technologies (more energy-efficient and environmentally friendly) for the achievement of higher process efficiency [12]. Furthermore, the choice of dried instead of fresh leaves has recently met strong opposition due to phytochemicals’ thermal decomposition or thermolabile compound oxidative condensation during plant drying [13,14].

Pulsed electric field (PEF) is an emerging eco-extraction technique of biologically active compounds (BACs). It is non-thermal with minimum energy requirements suitable for green solvents. PEF technology complies with environmental requirements for sustainable production systems [15]. The degree of the PEF’s efficiency in assisting the extraction of intracellular solutes from fresh plant materials relies on achieving a periodical pore formation to the lipid bilayer of the cell membrane, caused by the high voltage of PEF application. Ideally, by applying minimum specific energy through PEF application, electroporation occurs in such a way that components of interest inside the cell migrate outside the cell (where the solvent carries them away in solution) resulting in a mass transfer increase and, therefore, extraction yield improvement. The set of PEF parameters that require fine-tuning to enhance the extraction degree for a specific solid–liquid system include the strength of the electric field, the shape, width, and frequency of the pulse, and the duration of the application [5].

The most popular applications of PEFs include microorganism inactivation at high specific energy input levels [16,17], plant material pretreatment for further downstream processing at low to moderate specific energy input levels [18,19,20,21,22,23,24] or even the direct extraction of plant material [25]. The first authors that attempted to use PEF technology as the primary method for extraction were Brodelius et al. [26]. Even though, nowadays, attention has accumulated in fine-tuning PEF technology as a primary standalone extraction step for plant material BACs’ extraction depending on biomass properties, composition, and degree of comminution, there is still limited knowledge and plenty of room for discovery, innovation, and improvement on the plethora of plant materials and compounds of specific interest.

The scope of this work included investigation into the effect of PEFs on the solid–liquid static extraction of fresh SOLL using green solvents of gradual polarity, well-matched with the phenolic compounds’ polarity spectrum (pure ethanol (EtOH), pure water (H_2_O), and their mixtures at 25, 50, and 75% *v/v* concentrations). The pulse duration varied between two values under the same period and pulse type. The resulting extracts were evaluated against reference extracts obtained without PEF. The evaluation of the extraction efficiency was performed via determination of the content in total polyphenols (Folin–Ciocalteu method), individual phenolics (high-performance liquid chromatography, HPLC), volatile compounds (headspace solid-phase microextraction coupled to gas chromatography–mass spectrometry, HS-SPME/GC-MS) as well as resistance to oxidation (differential scanning calorimetry, DSC).

The novelty of this work lies upon the use of a non-thermal and eco-friendly technology, PEF, as the primary standalone extraction method for the extraction of *Salvia officinalis* L. BACs (including the thermolabile compounds) in green solvents (pure water, pure ethanol, and their mixtures), using fresh plant material instead of dried. The contribution to the scientific area of interest lies in the lack of similar experimental research on *Salvia officinalis* L.

## 2. Materials and Methods

### 2.1. Chemicals

The solvents used for chromatography were of HPLC grade. Formic acid (99%) and acetonitrile were obtained from Carlo Erba Co. (Val de Reuil, France). Sodium carbonate anhydrous (99%) and gallic acid monohydrate were from Penta Co. (Prague, Czech Republic), while Luteolin-7-*O*-glucoside, caffeic acid, and rosmarinic acid were from Sigma-Aldrich Co. (St. Louis, MO, USA). Ethanol (99.8%) and Folin–Ciocalteu reagent were acquired from Panreac Co. (Barcelona, Spain).

### 2.2. Plant Material, Handling and Sample Preparation

SOLL used in this study came from a single plant variety provided by a local greenhouse in Karditsa Region—Greece (at 39°21′53″ North and 21°56′21″ East and altitude of 105 m, according to Google Earth version 9.142.0.1, Google, Inc., Mountain View, CA, USA). The experiments’ series took place within five days (from 07 until 11 December 2020). The average temperature ranged between 7 °C and 15 °C, and the average relative humidity was 75%. The SOLL were delivered to the lab 5 min after their collection, early in the morning of each experimental day, and processed immediately.

After separation from the branches, SOLL were washed with water to discard impurities and then dried at ambient temperature (24 °C) using filter paper until no additional moisture was present on the leaves’ surface. Before each extraction trial, the leaves were pulverized (about 0.8 mm diameter) in a blender for 2 min, under identical shear input and batch quantities to ensure homogeneity of the pulverization outcome and minimum temperature rise. The latter resulted in high moisture content powders.

The selected solvent was added to the freshly cut SOLL immediately after grinding and the mixture was subsequently poured into the PEF treatment chamber. In all extraction runs, the raw material to solvent ratio was 1:3 (*w*/*v*), utilizing 16 g of SOLL and 48 mL of solvent. After 30 min of extraction, the suspensions were separated by decanting from the plant material, which was then discarded. The suspensions/extracts collected were transferred in a suitable Falcon tube and subjected to clarification via centrifugation (at 10,000× *g* at ambient temperature for 10 min) for immediate analysis. An infrared thermometer (GM300, Benetech, Shenzhen Jumaoyuan Science and Technology Co., Ltd., Shenzhen, China) was used to monitor the temperature of the treatment chamber contents before and after each extraction. The temperature increments due to the treatment never exceeded a ΔT of 1 °C.

### 2.3. Dry Matter Determination

Initially, an adequate amount of each sample batch of pulverized leaves were weighed and subsequently dried at 85 °C until constant weight using an oven (Binder BD56, Bohemia, NY, USA). The percentage of moisture and volatiles content was calculated as Equation (1)

(1) $$\%\mathrm{Moisture}\;\mathrm{and}\;\mathrm{volatiles}\;\mathrm{content}=\frac{\left(W_{BD}-W_{AD}\right)}{W_{BD}}\times 100$$

where *W_BD_* is the weight (g) of pulverized leaves before drying and *W_AD_* is the weight (g) of pulverized leaves after drying. The moisture and volatiles content of the leaves was about 80% (*w*/*w*). The dry matter (g) determination for each sample was calculated as Equation (2)

(2) $$\mathrm{Dry}\;\mathrm{matter}=W_{S}-\left(W_{S}\times \%\mathrm{Moisture}\;\mathrm{and}\;\mathrm{volatiles}\;\mathrm{content}\right)$$

where *W_S_* is the weight (g) of pulverized leaves without drying used as sample.

### 2.4. Pulsed Electric Field (PEF) Apparatus

An apparatus already presented by Pappas et al. [27] was utilized. In brief, a system comprised of a high voltage power generator (maximum voltage up to 25 kV), a 25 MHz function/arbitrary waveform generator, an electronic switch circuit (IGBTs), and a rectangular treatment chamber made of stainless steel with dimensions: 10 cm × 10 cm × 1 cm.

### 2.5. Extraction Parameters

The investigation boundaries incorporated the solvent used for extraction and the time for PEF treatment. In particular, pulse duration was 10 μs or 100 μs while the pulse period constant was 1000 μs, resulting in an energy input of 0.155 kJ·kg^−1^ and 1.55 kJ kg^−1^, or 2.52 × 10^−6^ KWh and 2.52 × 10^−5^ KWh, respectively. Five solvents were used; pure water, pure ethanol, and their mixtures at 25, 50, and 75% *v*/*v* concentrations. The criteria for the choice of the solvents were bound to our desire to utilize the minimum possible quantity of the green organic solvent (ethanol) in the aqueous mixture and our hypothesis that PEF technology would be revealed as beneficial for the extraction of BACs from fresh *Salvia officinalis* leaves using such a mixture. Reference samples were prepared in the very same manner but without the use of PEF, for comparison purposes. All extraction runs were performed in triplicates.

The electrical conductivity of solvents, the strength of the field, the treatment time, and the energy contribution (kJ·kg^−1^) determinations were measured as we have previously described [27]. Both PEF and reference samples were extracted for 30 min.

### 2.6. Determination of Total Polyphenol Content

The Folin–Ciocalteu assay was carried out as we have previously described [28]. Each sample was diluted to 1:50 (*v*/*v*) with deionized water. Next, 0.1 mL of each diluted sample was mixed with 0.1 mL Folin–Ciocalteu reagent into a 1.5 mL Eppendorf tube and was allowed to react for 2 min before the addition of 0.8 mL sodium carbonate (5% *w*/*v*). After 20 min of incubation in a water bath at 40 °C, the absorbance was obtained at 740 nm. The total polyphenol yield (Y_TP_) was determined as mg of gallic acid equivalents/g of dry weight (dw) (mg GAE g^−1^ dw) and based on a gallic acid calibration curve (10–80 mg L^−1^). A Shimadzu spectrophotometer (UV-1700, Shimadzu Europa GmbH, Duisburg, Germany) was used for the determinations.

### 2.7. HPLC

The method was adapted from Kaltsa et al. [29]. In brief, a Shimadzu liquid Chromatograph (CBM-20A) and a Shimadzu detector (SPD-M20A) were used. A Phenomenex Luna C18(2) (100 Å, 5 μm, 4.6 × 250 mm) (Phenomenex, Inc., Torrance, CA, USA) retained at 40 °C, a flow rate was 1 mL min^−1^, and an injection volume 20 μL were used. The mobile phases and the elution program used have been described previously [29]. Quantification calibration curves were prepared using three points (0, 10, and 50 mg mL^−1^), for caffeic acid (quantified at 320 nm, y = 0.000009x + 0.8755, R^2^ = 0.9986), rosmarinic acid (at 320 nm, y = 0.00002x + 0.3334, R^2^ = 0.9998), and luteolin-7-*O*-glucoside (at 345 nm, y = 0.00002x + 1.0794, R^2^ = 0.9980). The estimation of the total area was carried out at 245 nm and 350 nm.

### 2.8. Differential Scanning Calorimetry (DSC)

The DSC method used was adapted from Pappas et al. [27]. A Perkin Elmer Diamond DSC (PerkinElmer Inc, Shelton, CT, USA) was used. The antioxidant activity was determined using oxygen as the purge gas. The temperature program was as follows: hold at 40 °C for 1 min, heat from 40 to 200 °C (40 °C/min), and then heat from 20 to 580 °C (20 °C/min). The starting temperature of oxidation is the onset temperature of the oxidation peak (T_max_).

### 2.9. Volatile Compounds Analysis

The technique (HS-SPME/GC-MS) used was a modification of the method described by Hjelmeland et al. [30]. An SPME fiber coated with a layer of divinylbenzene/carboxen/polydimethylsiloxane (DVB/CAR/PDMS) (Supelco, Bellefonte, PA, USA), preconditioned for 30 min at 270 °C, was used. For HS-SPME extraction, 10 mL of the sample extract was placed in a 100 mL glass vial; 3 g of NaCl was added and sealed. The vial was kept at 40 °C during a 60 min period (10 min for equilibration + 50 min for extraction).

The analysis with GC-MS was carried out according to a modified method described by Hjelmeland et al. [30]. An Agilent Technologies (California, USA) Gas Chromatograph model 7890A equipped with a mass detector (5975C), and a capillary column Agilent J&W DB-1 (30 m × 320 μm × 0.25 μm) (California, CA, USA) were used. Helium was used as carrier gas at a flow rate of 1.5 mL min^−1^. The injector was operated in splitless mode at 240 °C. The temperature program was: 40 °C for 5 min, increased to 140 °C by 2 °C/min, and, finally, heated to 240 °C by 10 °C/min. Volatile compounds were identified by comparing their mass spectra with data from the integrated NIST 11 library (National Institute of Standards and Technology, Gaithersburg, MD, USA). The peaks were assigned when the similarity was above 80% and the component percentages were calculated as mean values from duplicate GC-MS analysis.

### 2.10. Statistical Analysis

All extraction series and spectrophotometric measurements were executed in triplicate. Microsoft Excel 2019 (Redmond, WA, USA) software was used for the statistical analysis of the results. ANOVA was used for the determination of the statistical significance (at *p* < 0.05) between mean values.

## 3. Results and Discussion

PEF applies millisecond- or even microsecond-long pulses of various voltages depending on the purpose of its application [31]. It was found that the higher increase of mass transfer rate was achieved by applying a PEF of 0.7–3.0 kV cm^−1^ and energy input of 1.0–20.0 kJ kg^−1^. During this study, we attempted to develop a PEF-assisted SOLL polyphenol extraction method under the scope of a green and sustainable standalone process. Moderate electric field intensity of 1 kV cm^−1^ and short pulses of 10 and 100 μs in a total processing time of 30 min were applied, while mixtures of EtOH–H_2_O in five different ratios were tested as extraction solvents. In particular, the gradual addition of ethanol to water (25% step gradient) was evaluated to determine whether the PEF effect could offset the increased recovery usually achieved using polar organic solvents in relation to water. EtOH and other polar organic solvents possess a good solubility and, thus, extractability for bioactive phytochemicals, but they must be removed after the end of the treatment, increasing the cost of the whole process. A possible reduction in the need for EtOH when using PEF can result in financial and environmental benefits. To the best of our knowledge, reported studies of SOLL extraction using PEF technology and aqueous organic solvents do not exist in the literature.

### 3.1. Total Phenol Content

According to the results, the highest percentage increase in total phenol (Y_TP_) between PEF and reference extracts was shown by the 25% EtOH solvent, after PEF with 100 μs pulse duration. The 75% EtOH and 100% EtOH solvents also showed significant increases, while pure water and 50% EtOH led to lower increases.

**Figure 1:**
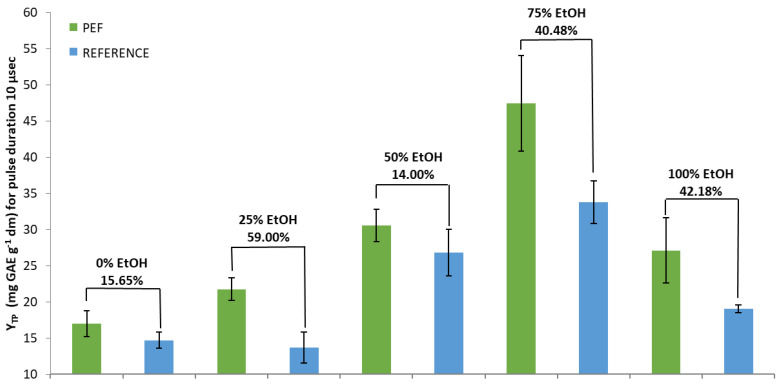
Y_TP_ for PEF-treated and reference extracts in five different tested solvents and a pulse duration of 10 μs. Y_TP_ is the total polyphenol yield.

In detail, regarding the pulse duration of 10 μs (Figure 1), the highest percentage increase in Y_TP_ between PEF and the reference sample obtained with the 25% EtOH solvent, was 59.00% (significant at *p* < 0.05). Specifically, Y_TP_ for the PEF sample was 21.76 mg GAE g^−1^ dw, while for the reference sample it was 13.68 GAE g^−1^ dw. The 75% EtOH and 100% EtOH solvents showed significant (*p* < 0.05) increases of 40.48% and 42.18%, respectively. The lowest increases presented with pure water and 50% EtOH were 15.65% and 14.00%, respectively. In these cases, PEF treated and reference samples showed no significant differences.

**Figure 2:**
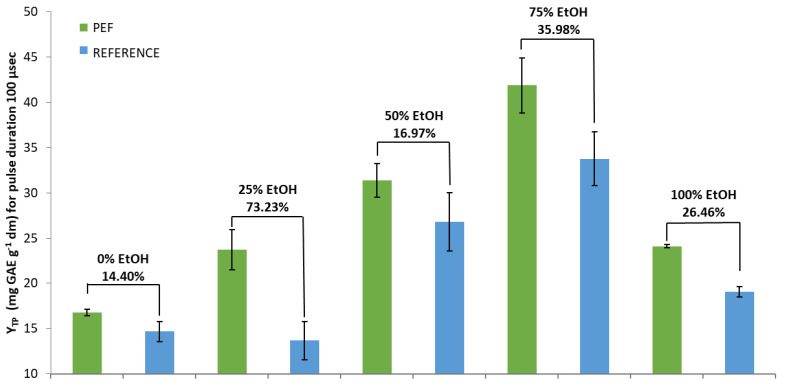
Y_TP_ for PEF treated and Reference extracts in five different tested solvents and a pulse duration of 100 μs. Y_TP_ is the total polyphenol yield.

The results of PEF treatment with a pulse duration of 100 μs are presented in Figure 2. The highest (significant at *p* < 0.05) percentage increase between PEF and the reference sample was achieved again with the 25% EtOH solvent (73.23%), and it was much higher (~24%) compared to that of the pulse duration of 10 μs. In addition, there were slightly higher increases concerning pure water and 50% EtOH (14.40% and 16.97%, respectively).

Although the highest percentage increases in Y_TP_ between PEF and reference extracts, for both pulse durations, were achieved with the addition of only 25% EtOH, the highest extraction rate was observed for the PEF-treated samples using 75% EtOH as solvent (Figure 1 and Figure 2). It seems the use of the PEF replenished part of the losses in the extraction rate when a solvent with low concentration of EtOH was used. Additionally, it was shown that longer-duration pulses appeared to deliver higher efficiency regarding the content in SOLL total polyphenols.

### 3.2. Differential Scanning Calorimetry (DSC)

As indicated by Batra et al. [32], this technique can determine the changes in the different physicochemical properties of compounds, which are shown by changes in the heat flow, and therefore, the oxidative stability of a sample can be evaluated through this technique [27]. DSC is the most widely used analytical technique for studies of physical characteristics and thermo-oxidative degradation of fats and oils, as well as their mixtures with herbal plant extracts, according to Kozłowska and Gruczyńska [33].

According to the results (Table 1), the maximum peak oxidation (T_max_) was 487 °C. The samples that produced this result (the highest antioxidant activity) were those with 75% EtOH and 10 μs pulse duration. This result was expected since the extracts in 75% ethanol were found to have the highest content in total polyphenols. The highest difference in the resistance to oxidation between PEF and reference extracts, expressed by the increase in oxidation temperature (significant at *p* < 0.05), was presented in the extracts produced with 25% EtOH. PEF extracts treated with 100 μs (25% EtOH) reached an average increase in oxidation temperature of 61.5% (in relation to reference extracts), while PEF extracts treated with a pulse duration of 10 μs reached a corresponding increase of 53.8%.

### 3.3. Volatile Compounds (VCs) Analysis

The results are shown in Table 2. The analysis was carried out only for the samples of 25% EtOH treated with PEF at 100 μs, which displayed the highest percentage increase in Y_TP_ and oxidation temperature between PEF and the reference extracts. The peak area obtained by HS-SPME/GC-MS was used to semi-quantify the concentration of different VCs.

Due to the large number of synonyms available, matching compounds by name tags was difficult, as was an accurate and thorough search using chemical IDs (e.g., CAS numbers). The identify compounds show strong MS similarity with library entries, without further information available.

The main components of SOLL previously identified were α- and β-thujone [34], α- and β-pinene, camphor and α-humulene [35], and 1,8-cineole, β-caryophyllene, camphene, myrcene, γ-terpinene, and *p*-cymene [36,37]. In our study, the principal VCs identified were eucalyptol, β-thujone, d- and l-camphor, 2-bornanol, borneol (endo-borneol), and l-borneol. These main compounds were identified in PEF-treated and reference extracts at about the same total percentage (65.51% and 67.58%, respectively). However, important differences appeared in the percentage of each of the above VCs between the differently treated samples. Specifically, d-camphor, borneol, and L-borneol showed an increase in PEF-treated extract, while eucalyptol, β-thujone, l-camphor, and 2-bornanol decreased. Differences also appeared in many other compounds (Table 2). Additionally, some compounds (*p*-cymen-8-ol, (−)-*trans*-pinocarveol, piperitenone, α-santalene, calarene, (+)-epizonarene, (+)-longifolene and (−)-γ-cadinene) appeared only in the PEF treated extract. The compounds *p*-cymen-8-ol and (−)-γ-cadinene were previously identified in *S. officinalis* L. leaves extracted by supercritical CO_2_ [38] or distillation–extraction [37]. Calarene and (−)-*trans*-pinocarveol were also previously reported [39,40].

The above results indicate that PEF effects can influence the aroma of SOLL extracts. Another study in line with the above conclusion is that of Sotelo et al. [41]. These authors studied the result of PEF technique on the flavor profile of red-fleshed sweet cherries and concluded that PEF-treated extracts produced higher amounts of volatile compounds that characterize the flavor, and that no adverse compounds appeared because low energy intensities were applied.

### 3.4. Extracts’ Characterization by HPLC

#### 3.4.1. Evaluation of PEF Effects Based on Extracts’ Total Area

Following the results regarding the estimation of Y_TP_ and oxidation temperature, the maximum percentage increase in total area between the PEF and reference extracts was reached by using 25% EtOH for both pulse durations. In particular, for pulse duration 10 μs, the increase was 72.83%, while for 100 μs it was 78.72%, both significant at *p* < 0.05 (see Figures S1 and S2, respectively, in Supplementary Materials). For pure water, there were minor changes (not significant—*p* > 0.05). In the case of 50% EtOH, significant increases (*p* < 0.05) of 19.42% for 10 μs and 13.44% for 100 μs appeared. Further addition of EtOH (75%) increased the percentage difference, in the case of the 10 μs pulse duration reaching a greater increase (significant at *p* < 0.05) in total areas than that of 100 μs (72.72% versus 25.46%, respectively). Finally, at 100% EtOH (pure EtOH), a significant increase in PEF-treated samples took place. The percentages were 52.73% versus 36.33% for 10 μs and 100 μs, respectively. It is worth mentioning at this point that there seems to be a clear trend indicating a gradual rise in the extraction yield when increasing the ethanol solvent content from 0% up to 75% EtOH, after which a drop takes place resulting in lower yields when pure ethanol was used.

#### 3.4.2. Polyphenolic Composition

For the estimation of PEF effects in the polyphenolic profile of SOLL, extracts in the optimum EtOH–H_2_O ratio (25% EtOH) were selected. The main compounds of SOLL found in this work are in line with previous findings [42,43]. As can be seen from the chromatogram of 25% EtOH and 100 μs PEF extract at 320 nm (Figure S3 in Supplementary Materials), four main compounds were identified that belong to the group of phenylpropanoids and flavones’ derivatives. Peak 1 was identified as caffeic acid and peak 4 as rosmarinic acid. The identification was based on the retention time (Figure S3 in Supplementary Materials) and absorption spectrum of the compounds and the corresponding reference substances. From our previous works, peaks 2 and 3 were identified as 6-hydroxy luteolin 7-*O*-glucoside [29] and luteolin 7-*O*-glucuronide [44], respectively. The amounts of the identified compounds, achieved in the extracts of reference samples processed with 25% EtOH, were 0.76 mg g^−1^ dw for 6-hydroxy luteolin 7-*O*-glucoside, 1.04 mg g^−1^ dw for luteolin 7-*O*-glucuronide, 0.17 mg g^−1^ dw for caffeic acid, and 0.37 mg g^−1^ dw for rosmarinic acid.

**Figure 3:**
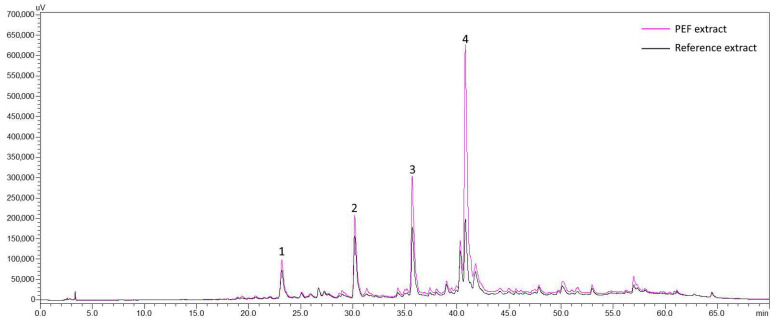
Overlay of chromatograms of PEF-treated and reference extracts at 320 nm with pulse duration 100 μs and extraction solvent 25% EtOH. Peak 1: Caffeic acid; Peak 2: 6-Hydroxy-luteolin-7-*O*-glucoside; Peak 3: Luteolin 7-*O*-glucuronide; Peak 4: Rosmarinic acid. Reference extract obtained without the application of PEF.

Quantification of the main identified components of SOLL was carried out in PEF extracts processed with the optimum EtOH–H_2_O ratio (25% EtOH) for both 100 μs and 10 μs pulse duration. The corresponding results prove the influence of PEF treatment (Table 3, Figure 3). As indicated by the estimation of Y_TP_, HPLC total area and oxidation temperature, the best performance in the extraction of identified phenolics was achieved by the pulses of 100 μs.

**Figure 4:**
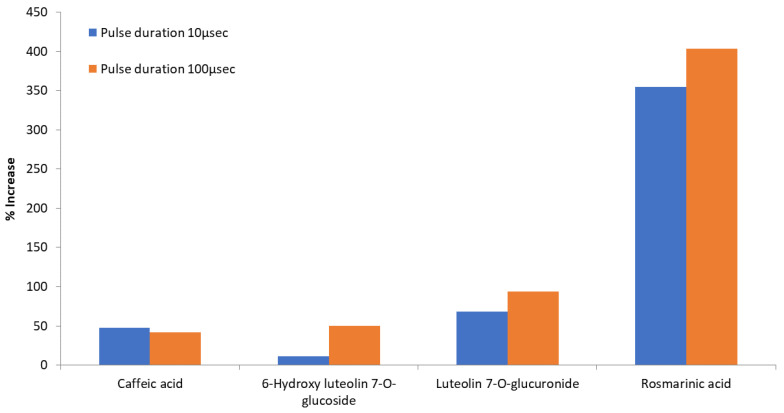
Comparison of the effect (% increase) of different pulse time durations on the extraction of individual phenolics in the same ethanol/water ratio (25% EtOH).

Specifically, extraction was enhanced for all examined compounds rising to a 403.12% increase for the 100 μs pulses and up to 354.61% for the 10 μs pulses. This remarkable and significant (*p* < 0.05) increase was shown for rosmarinic acid, achieving 1.85 mg g^−1^ dw in the optimum sample/extract (25% EtOH, 100 μs). It is well known that beyond the solvents and the extraction method chosen, the seasonality and the locality of a plant rule also the type and the levels of the components detected in plant extracts [5]. However, it is worth mentioning at this point that the above quantity of rosmarinic acid (1.85 mg g^−1^ dw achieved in the optimum extract) is significantly higher than the amount (0.45 mg g^−1^ dw) achieved by Bljajic et al. [45] using water ultrasound extraction for 30 min at high temperature (80 °C) and a higher liquid to solid ratio (10:1 mL g^−1^). For caffeic acid and 6-hydroxy-luteolin-7-*O*-glucoside, the significant (*p* < 0.05) increases for 100 μs pulses, reached 41.76% and 49.78%, respectively. An almost double increase, 93.49% (significant at *p* < 0.05), was reached for luteolin 7-*O*-glucuronide. It is obvious that both pulse durations succeeded in a permeabilization effect on the membranes and affected the extraction percentage of intracellular compounds from the cells. The big variance in percentage increases between the main compounds, for the same extraction conditions (Table 3, Figure 4), indicates the potential selectivity of this extraction method, a very important fact considering that the achievement of selective extraction is usually a tedious, time- and energy-consuming procedure. The main factors that possibly assist this selectivity are the molecular size and structure, the differences in cell membrane disintegration (such as pore size), the solubility of the extracted components, and the solvent’s polarity [31,46]. The differentiation of PEF processing parameters, as supported by the literature, also seem to support extraction selectivity. For 6-hydroxy luteolin 7-*O*-glucoside, in our study, the change in the pulse duration from 10 to 100 μs was followed by a higher increase in the levels of the metabolite, in comparison to the increase obtained for the three other identified metabolites (Table 3, Figure 4). A similar effect was observed during another study performed by our team regarding enhanced polyphenol extraction from olive leaves using PEF [27]. As in the current study, the effect of different duration pulses, 10 and 100 μs, was tested. The amounts of obtained metabolites varied depending on the applied pulse duration. Pulses of 100 μs favored oleuropein recovery, while 10 μs pulses favored the recovery of phenolic glucosides. Thus, the application of different PEF conditions (such as pulse duration of 10 or 100 μs) changes the extraction rate of each molecule promoting the selective extraction of various constituents from the SOLL.

In our work, PEF proved to be a green and effective extraction method. Although the comparison with other techniques cannot be direct because, as mentioned above, the levels of metabolites in each plant depend on both the seasonality and the origin of the plant, the proposed extraction technique can be characterized as efficient, since basic metabolites, identified in this particular study, appear to have been satisfactorily recovered [45]. The developed method can also be considered a green extraction technique because the optimal recovery of metabolites (between PEF- and not PEF-treated samples) was achieved in a low liquid-to-solid ratio (easier solvent removal), with only 25% addition of non-toxic organic solvent, low energy supply, and ambient temperature.

The results showed that PEF boosted the performance of SOLL extraction, revealing new targets for further improvement and insight into this technique. Further work, including process optimization (fine-tuning of important PEF parameters (i.e., number of pulses, etc.), is strongly advisable towards maximizing polyphenol concentration and extraction selectivity. The analysis of the role of solvent polarity in conjunction with PEF should be also evaluated.

## 4. Conclusions

This work is one of the very few studies that deal with PEF technique (a non-thermal and eco-friendly technology) as the primary standalone extraction step for freshly cut plant material BACs (including the thermolabile compounds) in green solvents. Even though BACs’ extraction depends on biomass properties, availability, composition, and degree of comminution, our findings for the specific plant material (*Salvia officinalis* L.) and solvent choice (ethanol, water, and their mixtures) revealed a substantial rise in the polyphenol concentration of the obtained extracts using different PEF conditions. The optimal detected PEF contribution, on the total polyphenol extractability (73.23% increase) and constituents of interest (up to 403.12% increase for specific metabolites) was presented by the 25% *v/v* aqueous ethanol solvent choice using a pulse of 100 μs for a 30 min extraction duration. The results were verified by the differential scanning calorimetry method, confirming our research target and initial hypothesis of achieving increased levels of extraction rate for the adding value components of the specific fresh plant material utilizing an aqueous green organic solvent with the minimum possible organic solvent content. PEF was proven to affect the final concentration and the composition of VCs in the extracts.

Despite the static nature of the specific extraction technique used in this study, which could be problematic for industrial applications (industries are favored by continuous production procedures), the above results denote that the PEF technique offers excellent potential for green selective extraction of biofunctional compounds from *Salvia officinalis* L. leaves. These compounds can serve in the preparation of high-quality functional foods or cosmetics.

## Figures and Tables

**Table 1:** Oxidation temperature (T_max_) of the various samples during DSC determination.

Extraction Solvent Synthesis	PEF Pulse Duration (μs)	T_max_ (°C) of PEF Treated Extract	T_max_ (°C) of Reference Extract	Increase (%)
0% EtOH	10	237± 3 ^1^	203 ± 2	16.7
	100	221 ± 2		8.9
25% EtOH	10	280 ± 3	182 ± 3	53.8
	100	294 ± 3		61.5
50% EtOH	10	350 ± 2	312 ± 5	12.2
	100	362 ± 4		16.0
75% EtOH	10	487 ± 7	387 ± 8	25.8
	100	462 ± 8		19.4
100% EtOH	10	294 ± 6	257 ± 7	14.4
	100	310 ± 7		20.6

^1^ Values are means of triplicate determinations ± Standard deviation.

**Table 2:** Percentage of volatile compounds determined in *Salvia officinalis* L. extracts (in 25% EtOH) by HS-SPME/GC-MS.

Compound	RT ^1^ (min)	Reference	PEF Treated (100 μs)	Compound	RT (min)	Reference	PEF Treated (100 μs)
*trans*, *trans*-2,4-Hexadienal	7.798	0.17	0.33	Piperitenone	34.651	nd	0.09
Benzaldehyde	9.907	0.05	Nd ^2^	Eugenol	36.322	0.02	0.06
Sabinene	12.190	0.03	nd	3-Carene	36.648	0.02	nd
1-Octen-3-ol	12.548	0.31	nd	α-Cubebene	37.305	0.12	0.10
β-Pinene	13.570	0.18	nd	L-Borneol acetate	38.244	0.04	nd
α-Terpinene	14.918	0.04	0.16	β-Cubebene	39.580	0.04	0.03
*p*-Cymene	15.099	0.16	0.30	α-Cubebene	39.980	0.01	nd
Eucalyptol	15.777	10.67 ^3^	3.35 ^3^	α-Gurjunene	40.753	0.09	nd
γ-Terpinene	17.686	0.30	0.23	Caryophyllene	41.089	0.56	0.06
*trans*-4-Thujanol	18.130	0.41	0.31	Aromandendrene	42.257	0.07	nd
*p*-Cymenene	19.233	0.07	0.06	δ-Cadinene	42.895	0.03	0.06
2-Carene	19.621	0.11	nd	Humulene	43.034	0.11	nd
α-Thujone	20.110	3.02	1.44	β-Copaene	43.577	0.04	0.04
β-Thujone	21.139	11.07	4.10	δ-Cadinene	44.452	0.02	nd
D-Camphor	22.397	16.05	19.82	Germacrene D	44.641	0.15	0.21
DL-Camphor	22.437	1.38	nd	γ-Cadinene	44.952	0.08	0.07
L-Camphor	22.611	8.23	nd	α-Cadinene	45.147	0.07	0.17
Camphene	22.689	0.47	nd	α-Elemene	45.574	0.07	nd
L-Camphene	23.100	0.71	nd	Epizonarene	45.669	0.08	0.13
D-Pinocamphone	23.388	0.07	nd	α-Muurolene	46.088	0.10	0.18
Isoborneol	23.666	0.17	nd	γ-Muurolene	46.665	0.19	0.22
2-Bornanol	24.350	7.00	2.33	*cis*-Calamenene	46.822	0.07	0.06
Borneol	24.522	3.91	9.56	γ-Elemene	47.050	0.74	1.26
L-Borneol	24.898	10.65	26.35	α-Santalene	47.216	nd	0.81
Borneol	24.970	3.43	nd	δ-Cadinene	47.413	0.30	0.41
*p* -Cymen-8-ol ^4^	25.325	nd	0.17	1,4-Cadinadiene	47.774	0.03	nd
Terpinen-4-ol	25.426	1.21	1.55	α-Calacorene	47.890	0.03	nd
4-Carene	25.787	0.15	0.20	(-)-α-Cadinene	48.106	0.02	0.06
α-Terpineol	26.177	0.57	1.44	Espatulenol	49.771	0.07	0.12
Myrtenol	26.569	0.20	0.52	Caryophyllene oxide	49.928	0.18	0.11
(-)-*trans*-Pinocarveol	26.669	nd	0.10	Diethyl phthalate	50.049	0.10	0.08
*cis*-Carveol	27.927	0.05	0.10	(+)-γ-Gurjunene	50.714	0.38	0.36
*cis*-3-Hexenyl valerate	29.787	0.07	nd	α-Guaiene	51.309	0.07	nd
*trans*-2-Hexenyl valerate	30.328	0.05	nd	Calarene	53.430	nd	0.19
α-Ocimene	30.871	0.07	0.22	(+)-Epizonarene	53.683	nd	0.04
6-Oxocamphor	31.162	0.17	0.55	β-Guaiene	53.714	0.05	nd
Bornyl acetate	32.491	1.65	0.43	Alloaromadendrene	54.477	0.04	nd
Sabinyl acetate	32.980	0.21	0.07	(+)-Longifolene	54.716	nd	0.04
Thymol	33.322	0.11	0.23	(-)-γ-Cadinene	56.080	nd	0.51
Carvacrol	33.789	0.42	0.39	(-)-α-Amorphene	56.134	0.41	nd
Reference extract Total = 87.97%				PEF treated extract Total = 79.80%			

^1^ RT: Retention Time; ^2^ nd: (*m*/*z*) spectra were not detected; ^3^ Blue and green colors denote the highest concentration of compounds; ^4^ Red color denotes compounds identified only in PEF treated samples.

**Table 3:** Major compounds (mg g^−1^ dw) of *Salvia officinalis* L. PEF treated (pulse duration of 100 μs) and reference extracts, all prepared with 25% ethanol.

PEF Pulse Duration	Compound	PEF Treated Extract	Reference Extract	Increase (%)
100 μs	Caffeic acid	0.24 ± 0.04 ^1^	0.17 ± 0.04	41.76
	6-Hydroxy luteolin 7-*O*-glucoside ^2^	1.14 ± 0.28	0.76 ± 0.28	49.78
	Luteolin 7-*O*-glucuronide ^2^	2.01 ± 0.32	1.04 ± 0.24	93.49
	Rosmarinic acid	1.85 ± 0.82	0.37 ± 0.35	403.12
10 μs	Caffeic acid	0.25 ± 0.04	0.17 ± 0.04	47.37
	6-Hydroxy luteolin 7-*O*-glucoside ^2^	0.85 ± 0.07	0.76 ± 0.28	11.49
	Luteolin 7-*O*-glucuronide ^2^	1.75 ± 0.35	1.04 ± 0.24	68.01
	Rosmarinic acid	1.67 ± 1.01	0.37 ± 0.35	354.61

^1^ Values are means of triplicate determinations ± Standard deviation; ^2^ 6-hydroxy luteolin 7-*O*-glucoside and luteolin 7-*O*-glucuronide were quantified as Luteolin 7-*O*-glucoside.

## Data Availability

The original contributions presented in the study are included in the article/Supplementary Materials. Further inquiries can be directed to the corresponding author/s.

## Supplementary Materials

The following are available online at https://www.mdpi.com/article/10.3390/foods10092014/s1, Figure S1: HPLC total area for PEF and Reference extracts in five different tested solvents and a pulse duration of 10 μs, Figure S2: HPLC total area for PEF and Reference extracts in five different tested solvents and a pulse duration of 100 μs, Figure S3: Overlay of chromatograms of extract and reference compounds at 320 nm after PEF with pulse duration 100 μs and extraction solvent 25% EtOH. Peak 1: Caffeic acid; Peak 2: 6-Hydroxy-luteolin-7-*O*-glucoside; Peak 3: Luteolin 7-*O*-glucuronide; Peak 4: Rosmarinic acid.
