# Supplementary material for: Pulsed Electric Field and Salvia officinalis L. Leaves: A Successful Combination for the Extraction of High Value Added Compounds
Source: Foods. 2021 Aug 27;10(9):2014. doi: 10.3390/foods10092014 (PMC8469738; doi:10.3390/foods10092014)
Supplement: Supplementary file 1 [file foods-10-02014-s001.zip › foods-1314210-supplementary.pdf]

Supplementary material

Figure Legends

Figure S1. HPLC total area for PEF and Reference extracts in five different tested solvents and a pulse duration of 10 μsec.

Figure S2. HPLC total area for PEF and Reference extracts in five different tested solvents and a pulse duration of 100 μsec.

Figure S3. Overlay of chromatograms of extract and reference compounds at 320 nm after PEF with pulse duration 100 μsec and extraction solvent 25% EtOH. Peak 1: Caffeic acid; Peak 2: 6-Hydroxy-luteolin-7-O-glucoside; Peak 3: Luteolin 7-O-glucuronide; Peak 4: Rosmarinic acid.

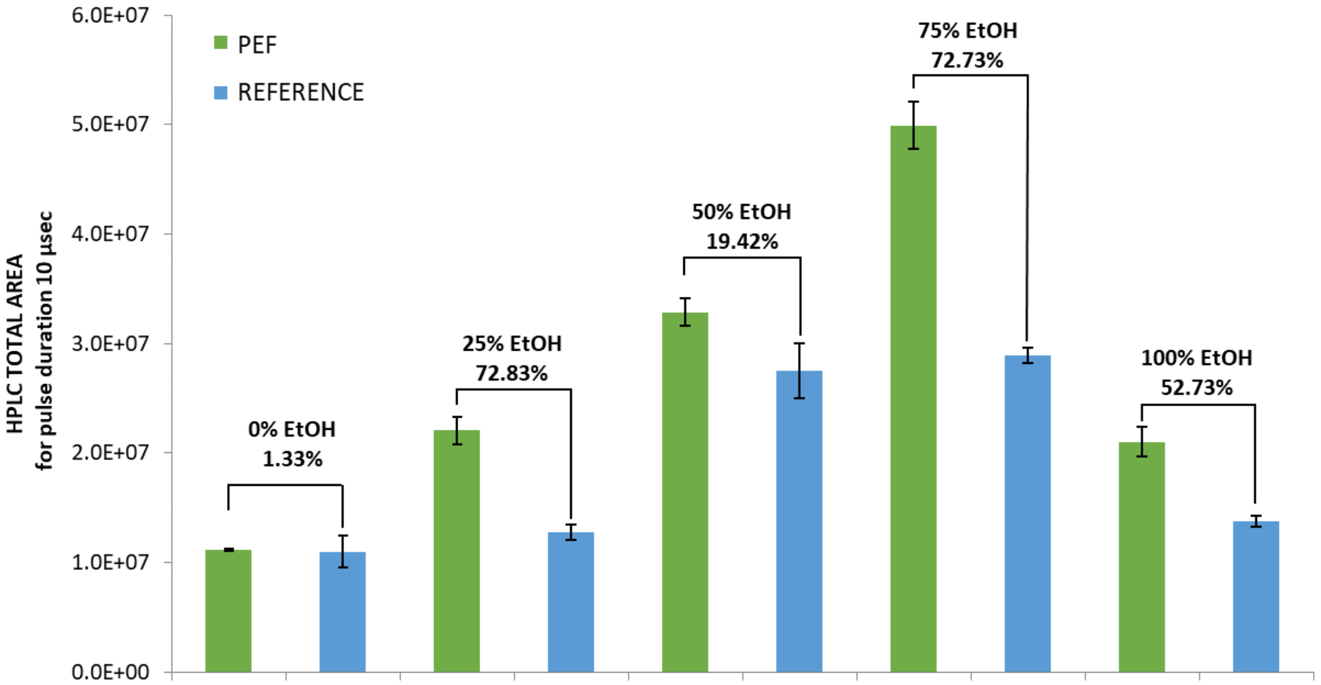

Figure S1. HPLC total area for PEF and Reference extracts in five different tested solvents and a pulse duration of 10 μsec.

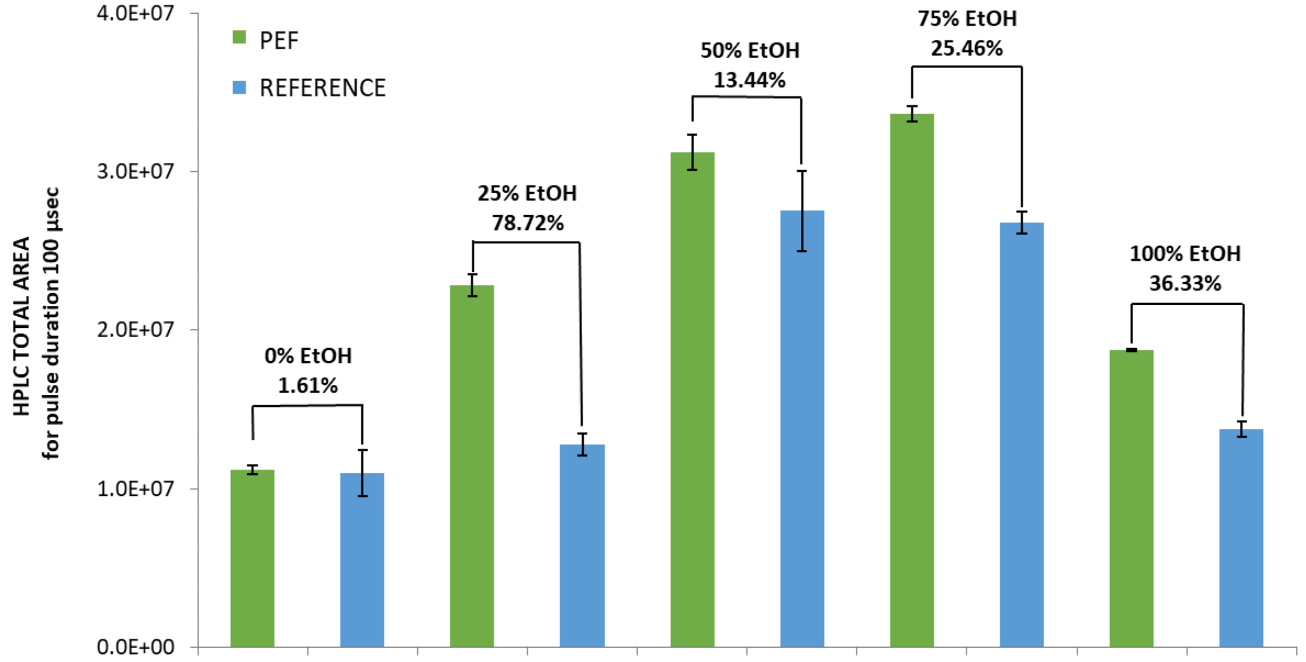

Figure S2. HPLC total area for PEF and Reference extracts in five different tested solvents and a pulse duration of 100 μsec.

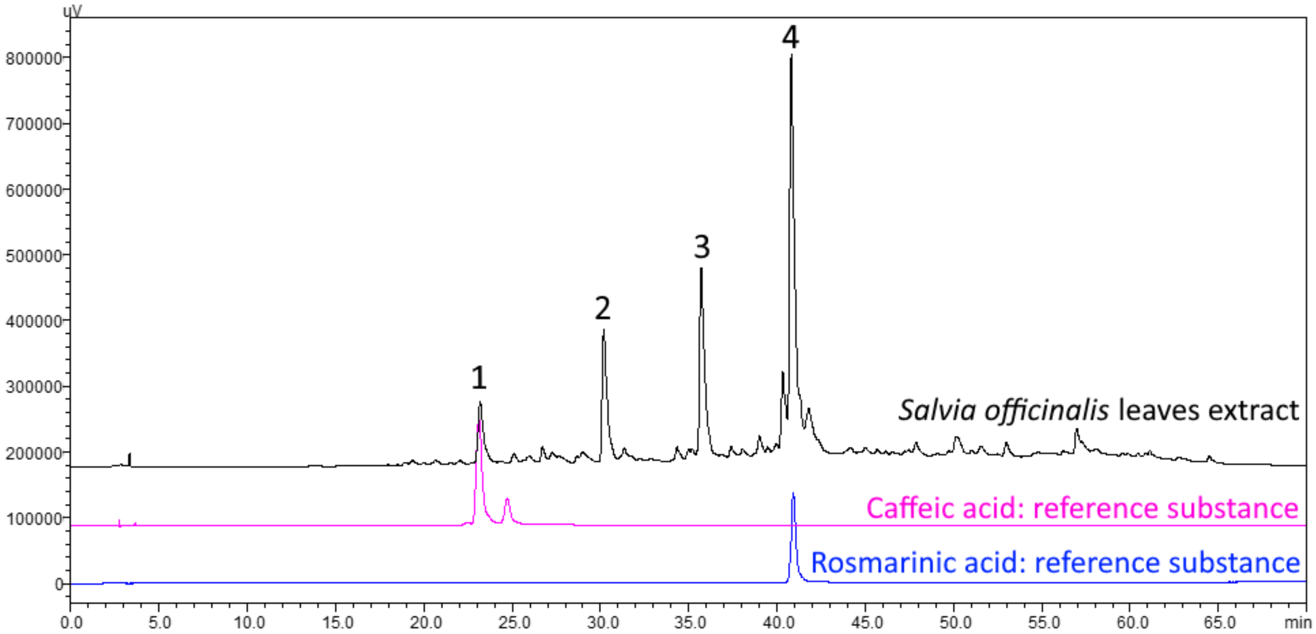

Figure S3. Overlay of chromatograms of extract and reference compounds at 320 nm after PEF with pulse duration 100 μsec and extraction solvent 25% EtOH. Peak 1: Caffeic acid; Peak 2: 6-Hydroxy-luteolin-7-O-glucoside; Peak 3: Luteolin 7-O-glucuronide; Peak 4: Rosmarinic acid.
